# Mesoporous Mn-substituted Mn_x_Zn_1−x_Co_2_O_4_ ternary spinel microspheres with enhanced electrochemical performance for supercapacitor applications

**DOI:** 10.1038/s41598-024-58822-0

**Published:** 2024-05-19

**Authors:** Tarekegn Heliso Dolla, Isiaka Ayobamidele Lawal, Gizachew Wendimu Kifle, Samuel Chufamo Jikamo, Thabo Matthews, Nobanathi Wendy Maxakato, Xinying Liu, Mkhulu Mathe, David Gordon Billing, Patrick Ndungu

**Affiliations:** 1 Institute for Catalysis and Energy Solutions (ICES), University of South Africa (UNISA), Florida, 1709 South Africa; 2 Department of Biotechnology and Chemistry, Vaal University of Technology, Vanderbijlpark, 1911 South Africa; 3 Department of Chemistry, Wolaita Sodo University, P. O. Box 138, Wolaita Sodo, Ethiopia; 4 Department of Chemical Sciences, University of Johannesburg, Doornfontein, 2028 South Africa; 5 Molecular Sciences Institute, School of Chemistry, University of the Witwatersrand, Johannesburg, South Africa; 6 Department of Chemistry, University of Pretoria, Pretoria, 0001 South Africa

**Keywords:** Ternary spinel oxides, Supercapacitors, Coprecipitation, Microspheres, Energy storage, Chemistry, Materials science, Nanoscience and technology

## Abstract

Extensive investigations have been carried out on spinel mixed transition metal oxide-based materials for high-performance electrochemical energy storage applications. In this study, mesoporous Mn-substituted Mn_x_Zn_1−x_Co_2_O_4_ (ZMC) ternary oxide microspheres (x = 0, 0.3, 0.5, 0.7, and 1) were fabricated as electrode materials for supercapacitors through a facile coprecipitation method. Electron microscopy analysis revealed the formation of microspheres comprising interconnected aggregates of nanoparticles. Furthermore, the substitution of Mn into ZnCo_2_O_4_ significantly improved the surface area of the synthesized samples. The electrochemical test results demonstrate that the ZMC3 oxide microspheres with an optimal Mn substitution exhibited enhanced performance, displaying the largest specific capacitance of 589.9 F g^−1^ at 1 A g^−1^. Additionally, the ZMC3 electrode maintained a capacitance retention of 92.1% after 1000 cycles and exhibited a significant rate capability at a current density of 10 A g^−1^. This improved performance can be ascribed to the synergistic effects of multiple metals resulting from Mn substitution, along with an increase in the surface area, which tailors the redox behavior of ZnCo_2_O_4_ (ZC) and facilitates charge transfer. These findings indicate that the incorporation of Mn into mixed transition metal oxides holds promise as an effective strategy for designing high-performance electrodes for energy storage applications.

## Introduction

With the development of various portable electronic devices, the emergence of electric vehicles, and the need for large-scale energy storage from renewable sources, there has been a sharp increase in the demand for advanced energy storage technologies^1^. Energy storage devices play a crucial role in meeting these demands. Supercapacitors (SCs) have attracted considerable attention as energy storage devices because of their long cycle life, high power density, fast charge/discharge rate, and low manufacturing cost^2,3^. Current commercial electrochemical capacitors are based on carbon materials as electrode materials, which store charge by charging the electrochemical double layer at the electrode–electrolyte interface. However, it does not meet the increasing demand owing to its low energy density^2,4–6^. Pseudocapacitors, which exhibit fast surface redox reactions, have emerged as devices with high energy densities owing to their high theoretical capacity^7^. Transition metal oxides have been extensively explored as pseudocapacitive and battery type electrode materials because of their excellent theoretical specific capacities and fast charge/discharge abilities^8,9^. However, these materials suffer from sluggish electrochemical reaction kinetics and low electrical conductivity, which limit their practical applicability^10^. Consequently, significant efforts have been devoted to the development of transition metal oxides with tunable nanostructures and compositions to improve their electrochemical performances^11–13^.

In recent years, spinel-type multimetal oxides have been extensively investigated as high-performance electrode materials for supercapacitors^14,15^. This is due to the multiple oxidation states, better electrochemical performance, and improved electrical conductivity compared to their single-metal counterparts^16–18^. Particularly, spinel cobaltites belonging to the MCo_2_O_4_ class with a single-phase structure have been extensively investigated as electrode materials owing to their enhanced electrochemical performance^19,20^. What sets cobalt-based metal oxides apart from other spinel-type materials is their distinctive battery-like behavior, primarily attributed to the generation of oxyhydroxides during charge storage, resulting in a notably high theoretical specific capacity^21^. Furthermore, cobalt exhibits remarkable features by enhancing the electronic conductivity of metal oxides, making it an appealing choice for such applications. The combination of different cations to form multimetallic spinel oxides and the ability to adjust the stoichiometric/non-stoichiometric compositions of these oxides offer great opportunities to manipulate their electrochemical properties^22–24^. Thus, strategies based on the substitution of metal cations in host metal oxides and the rational design of nanostructures and microstructures have been widely employed to develop high-performance mixed-transition-metal oxide electrodes. In this regard, numerous studies of transition metal oxides composed of multiple metals, including Mn_1−x_Ni_x_Co_2_O_4_^25^, Zn–Mn–Co ternary oxide nanoneedles^26^, Zn–Ni–Co ternary oxide microspheres^27^, Cu–Zn–Co oxide nanoflakes^28^, Mn-doped NiCo_2_O_4_^29^, mesoporous MnZnFe_2_O_4_ nanoneedles^30^, and mixed ternary transition metal ferrites^31^ have been reported. These investigations highlight the synergistic effect of incorporating multiple transition metals and the tunability of their compositions to enhance the electrochemical performance of transition metal oxides as supercapacitor electrodes. Therefore, metal cation substitution is a feasible approach for tuning the chemical composition, electronic structure, electrical conductivity, and electrochemical properties of the electrode materials.

Manganese is regarded as a desirable metal for substitution or doping in host transition metal oxide electrode materials because of its favorable characteristics such as multiple oxidation states, abundance in the Earth's crust, and environmental friendliness^32–34^. In most Mn-containing electrode materials, Mn^2+^/ Mn^3+^/Mn^4+^ is observed, which is responsible for the enhanced carrier mobility, improved specific capacitance and cycling stability, and adjustable surface electrolyte ion adsorption/desorption energy^29,35–39^. Considering these advantages, numerous studies have focused on the substitution or doping of Mn ions into transition metal oxides to achieve improved electrochemical performance. For example, Sharif et al.^40^ synthesized Ni_1−x_Mn_x_Fe_2_O_4_ nanoparticles and investigated the effect of the incremental substitution of Ni with Mn on the electrochemical performance of supercapacitor electrodes. They found that the specific capacitance of the prepared Ni_1−x_Mn_x_Fe_2_O_4_ nanoparticles increased with an increase in the Mn content. Mary et al.^41^ reported Mn-doped ZnCo_2_O_4_ with an optimal 10 wt% Mn doping and intact structure and showcased an improved specific capacitance. In another study, Zhang et al. prepared a series of Mn-substituted NiCo_2_O_4_ (MNCO) nanowires using a hydrothermal annealing strategy and varying the Mn/Ni molar ratio^33^. The results indicate that the Mn^3+^ substituting of Ni^3+^ in NiCo_2_O_4_ optimizes the electronic structure, enhances the charge transfer kinetics, and improves the electrochemical activity of NiCo_2_O_4_. Recently, Sun et al.^42^ reported Mn in situ isomorphism-doped CoCo_2_O_4_ porous nanowires prepared using a microwave-assisted hydrothermal process. The Mn-incorporated porous nanowires exhibited an improved capacity and a broadened voltage window. The Mn^2+^ and Mn^3+^ dopants in CoCo_2_O_4_ provide additional redox sites by forming reversible Mn^4+^, thereby enhancing the energy storage capacity. Therefore, Mn doping or substitution in transition metal oxides can be considered an effective strategy to improve their electrochemical performance for supercapacitor applications.

In this study, we prepared ternary spinel microspheres of Mn-substituted Mn_x_Zn_1−x_Co_2_O_4_ (ZMC) through a straightforward coprecipitation route followed by calcination based on our previously reported study^43,44^. Our investigation is targeted on the impact of manganese (Mn) substitution on both the structural and morphological characteristics of these microspheres, as well as their electrochemical performance. On substitution of Mn, a significant change is observed on the size of the ZMC microspheres. Specifically, their size grew, and their specific surface area expanded, reaching a remarkable 56.27 m^2^ g^−1^ for ZMC3, surpassing that of ZC (17.79 m^2^ g^−1^). The electrochemical evaluations revealed that at the optimal Mn substitution, the porous ZMC3 microspheres achieved an outstanding specific capacitance of 589.9 F g^−1^ at a current density of 1 A g^−1^. Furthermore, they exhibited improved cycling stability, retaining 92.1% of the initial specific capacitance after 1000 cycles, outperforming other compositions. These findings demonstrate the effectiveness of Mn substitution as a viable strategy for enhancing the electrochemical performance of transition metal oxide electrodes for energy-storage applications. Furthermore, this work contributes to the expanding family of ternary spinel oxides among multimetal oxide nanostructures.

## Experimental

### Synthesis of the mesoporous Mn_x_Zn_1−x_Co_2_O_4_ microspheres

All chemicals used in this study were of analytical grade and were employed without any additional purification steps. The materials were fabricated using the coprecipitation method as previously reported by our research group, with minor adjustments^41^. In this procedure, each sample was created by dissolving the requisite quantities of metal acetate precursors, specifically zinc acetate, manganese acetate, and cobalt acetates, in a mixture of distilled water and ethanol (in a 10:1 ratio), resulting in a total volume of 230 mL. For instance, to synthesize Mn_0.5_Zn_0.5_Co_2_O_4_ microspheres (x = 0.5), 1 mmol of Mn(Ac)_2_·4H_2_O, 1 mmol of Zn(Ac)_2_·2H_2_O, and 4 mmol of Co(Ac)_2_·4H_2_O were dissolved in a mixture of 21 mL of ethanol and 210 mL of distilled water under vigorous stirring. Another solution was prepared by dissolving 60 mmol of NH_4_HCO_3_ in 230 mL of distilled water, followed by slow addition to the metal precursor solution with continuous stirring. The resulting mixture was heated to 45 °C and held at that temperature for 9 h with continuous stirring. A pale pink precipitate formed, which was then collected through filtration, thoroughly washed with distilled water and ethanol, and subsequently dried at 60 °C overnight. The resulting carbonate precursor was subjected to heat treatment in an air environment at 600 °C for 5 h, employing a temperature ramp of 2 °C per minute, ultimately yielding a black powder. The as-prepared samples of Mn_x_Zn_1−x_Co_2_O_4_ (ZMC) with x = 0, 0.3, 0.5, 0.7, and 1 were designated as ZC, ZMC1, ZMC2, ZMC3, and MC, respectively.

### Characterization of the samples

X-ray diffraction (XRD) was obtained using a Bruker D2 Phaser X-ray diffractometer equipped with Cu K radiation (λ = 0.709321 Å) at 30 kV. The scan spanned from 10° to 70° 2θ in increments of 0.0260°. Transmission electron microscopy (TEM) analysis was conducted utilizing a JEM-2100 transmission electron microscope, while scanning electron microscopy (SEM) analysis employed a FEI Nova Nano SEM 450 scanning electron microscope. The N_2_ adsorption/desorption experiments were performed to determine BET surface area and pore size distribution using a Micromeritics ASAP 2020 surface area and porosity analyzer. Prior to the experiment, the sample was subjected to outgassing at 150 °C for 5 h under a nitrogen gas atmosphere. BET surface areas were determined from adsorption data within a relative pressure range of 0.05–0.30, and the total pore volume was calculated based on the quantity of N2 vapor adsorbed at a relative pressure of 0.99. The pore size distributions were derived from the desorption branches of the isotherms.

### Fabrication of electrodes and electrochemical performance test

The electrodes were fabricated as follows: the active material, activated carbon, and polyvinylidene fluoride (PVDF) binder were mixed in an 80:10:10 (wt. %) ratio. A mixture slurry was made using N-Methyl-2-pyrrolidone (NMP), which was coated on a nickel foil current collector of (1 × 1 cm) 1.6 mm thickness (produced by MTI corporation) and dried for 4 h to remove the solvent. The weight of active material loaded was in the range of 1–1.5 mg. Cyclic Voltammetry (CV), chronopotentiometry (CP), and Electrochemical Impedance spectroscopy (EIS) were performed using a Gamry potentiostat/galvanostat, employing a standard 3-electrode cell configuration with a platinum wire as the counter electrode and Ag/AgCl as the reference electrode. The measurements were performed using an aqueous 2 M KOH electrolyte under ambient conditions.

## Results and discussion

### Structure and morphology characterizations

**Scheme 1:**
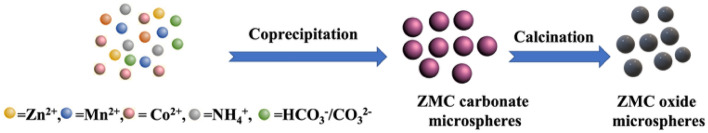
Schematic illustration of the formation process of ZMC oxide microspheres.

**Figure 1:**
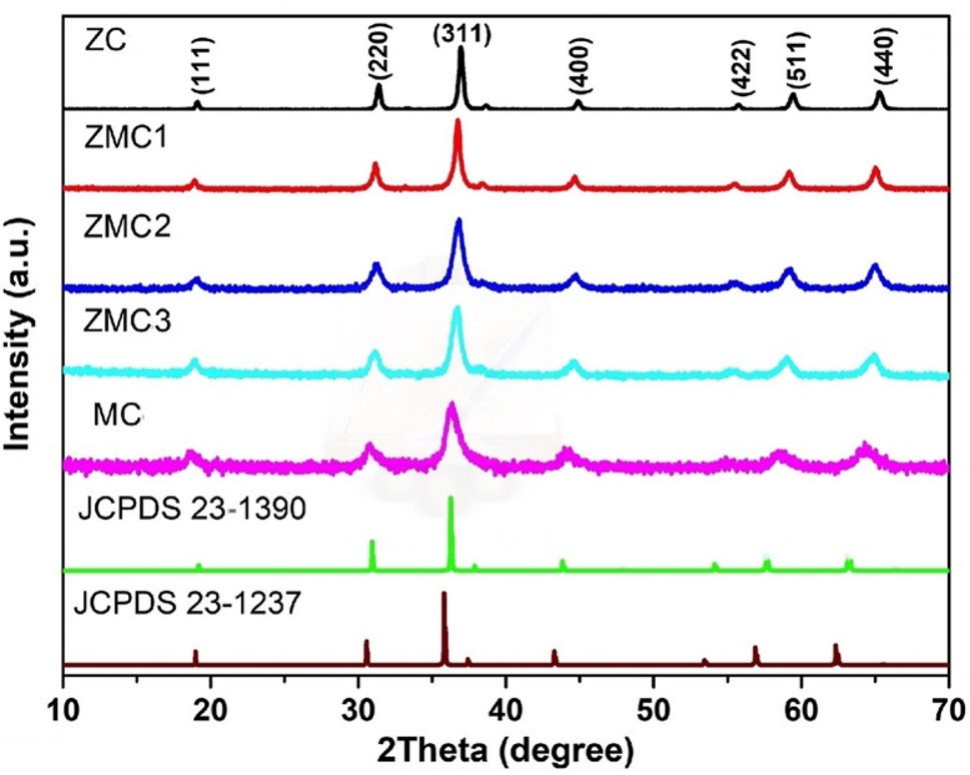
The XRD patterns of the as-synthesized ZMC oxide microspheres, along with those of the reference materials ZnCo_2_O_4_ (JCPDS no. 23-1390) and MnCo_2_O_4_ (JCPDS no. 23-1237).

Mesoporous Mn_x_Zn_1−x_Co_2_O_4_ (ZMC) ternary oxide microspheres were synthesized via a two-step process, as depicted in Scheme 1. Initially, ZMC carbonate microspheres were formed through a simple coprecipitation method using NH_4_HCO_3_ as the precipitating agent. Subsequently, the ZMC carbonate microspheres were calcined at 600 °C, resulting in the formation of the ZMC oxide microspheres. This calcination process facilitated the creation of numerous pores by eliminating gaseous H_2_O and CO_2_ and promoting interactions between the constituent nanoparticles, thereby resulting in the formation of porous microspheres. To examine the crystalline structure, X-ray diffraction (XRD) was performed in the range of 10°–70° 2θ, as shown in Fig. 1. The XRD patterns of all the samples show the main characteristic peaks at 18.5°, 31.3°, 36.2°, 44.3°, 59.1°, and 64.3°, which are well indexed to the (111), (220), (311), (422), (511), and (440) crystallographic planes, respectively. The XRD patterns were assigned to ZnCo_2_O_4_ (JCPDS no. 23-1390) and/or MnCo_2_O_4_ (JCPDS no. 23-1237), both with a cubic spinel structure and crystallizing in the *Fd-3 m* space group (227). No discernible diffraction peaks originating from impurities are observed in any of the XRD patterns. These results confirmed the successful formation of ternary spinel ZMC oxides. In comparison to the pure ZnCo_2_O_4_, the most intense peak of the (311) plane in Mn_x_Zn_1−x_Co_2_O_4_ displayed a slight shift towards a lower angle. This shift indicated the successful incorporation of Mn atoms into the crystal lattice of ZnCo_2_O_4_ through doping. The peaks of the Mn substituted samples are also observed to broaden with increasing Mn concentration compared to the ZnCo_2_O_4_. The Rietveld analysis was utilized to calculate the lattice parameters. It was observed that ZnCo_2_O_4_ exhibits a lattice parameter of a = 8.0874 Å, which closely aligns with the reported values. When Mn ions were introduced as substitutes for Zn^2+^, the lattice parameter increased in comparison to pure ZnCo_2_O_4_ due to the slightly larger ionic size of Mn ions (0.740 Å)^45^. This increase in lattice parameter demonstrates a clear and linear trend with the gradual doping of Mn ions, confirming the successful substitution of Zn by Mn ions. Additionally, the crystallite size is observed to decrease from 51 to 19 nm with increasing Mn concentration.

**Figure 2:**
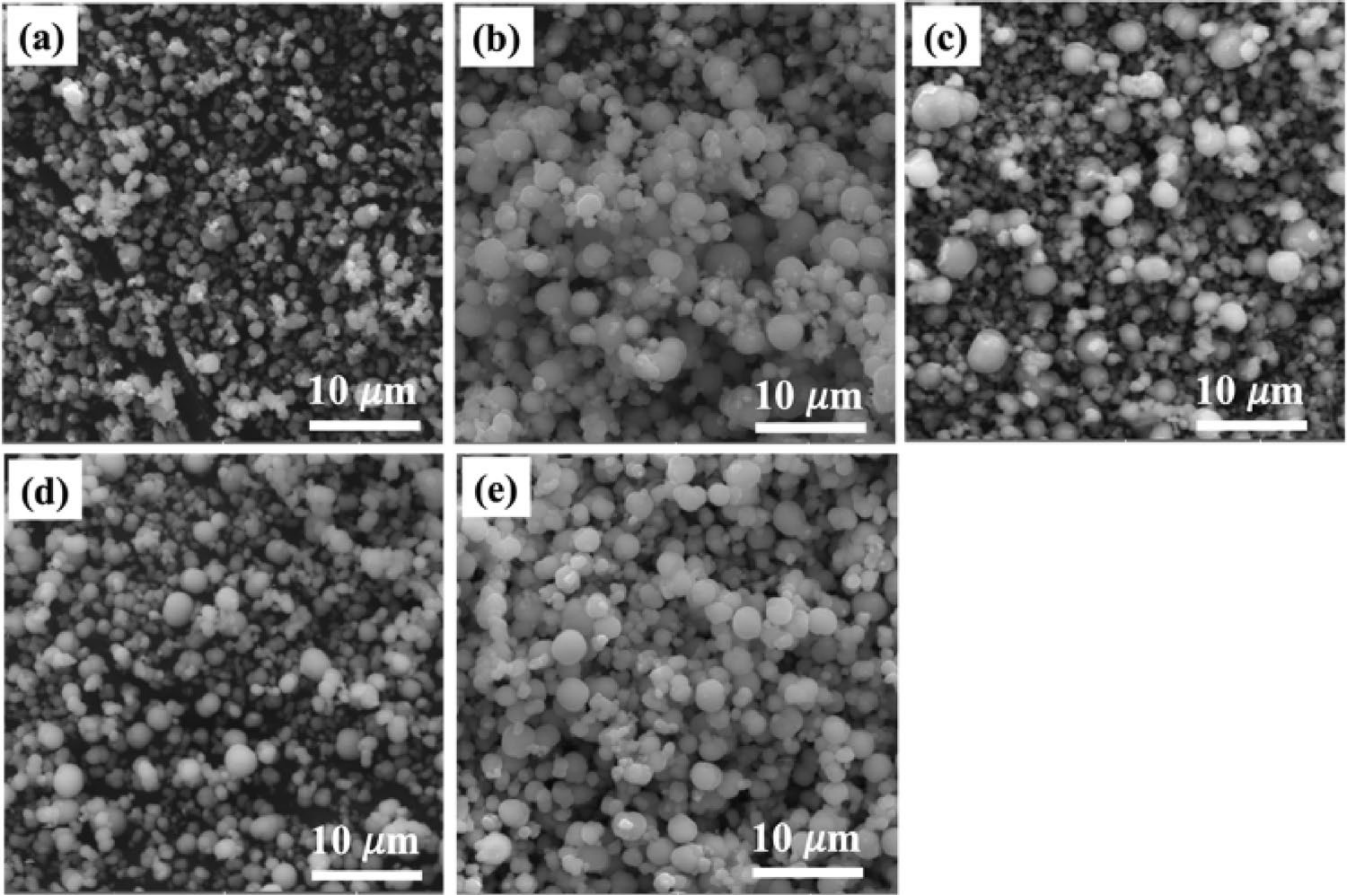
SEM image of ZMC microspheres (**a**) ZC, (**b**) ZMC1, (**c**) ZMC2, (**d**) ZMC3, and (**e**) MC.

**Figure 3:**
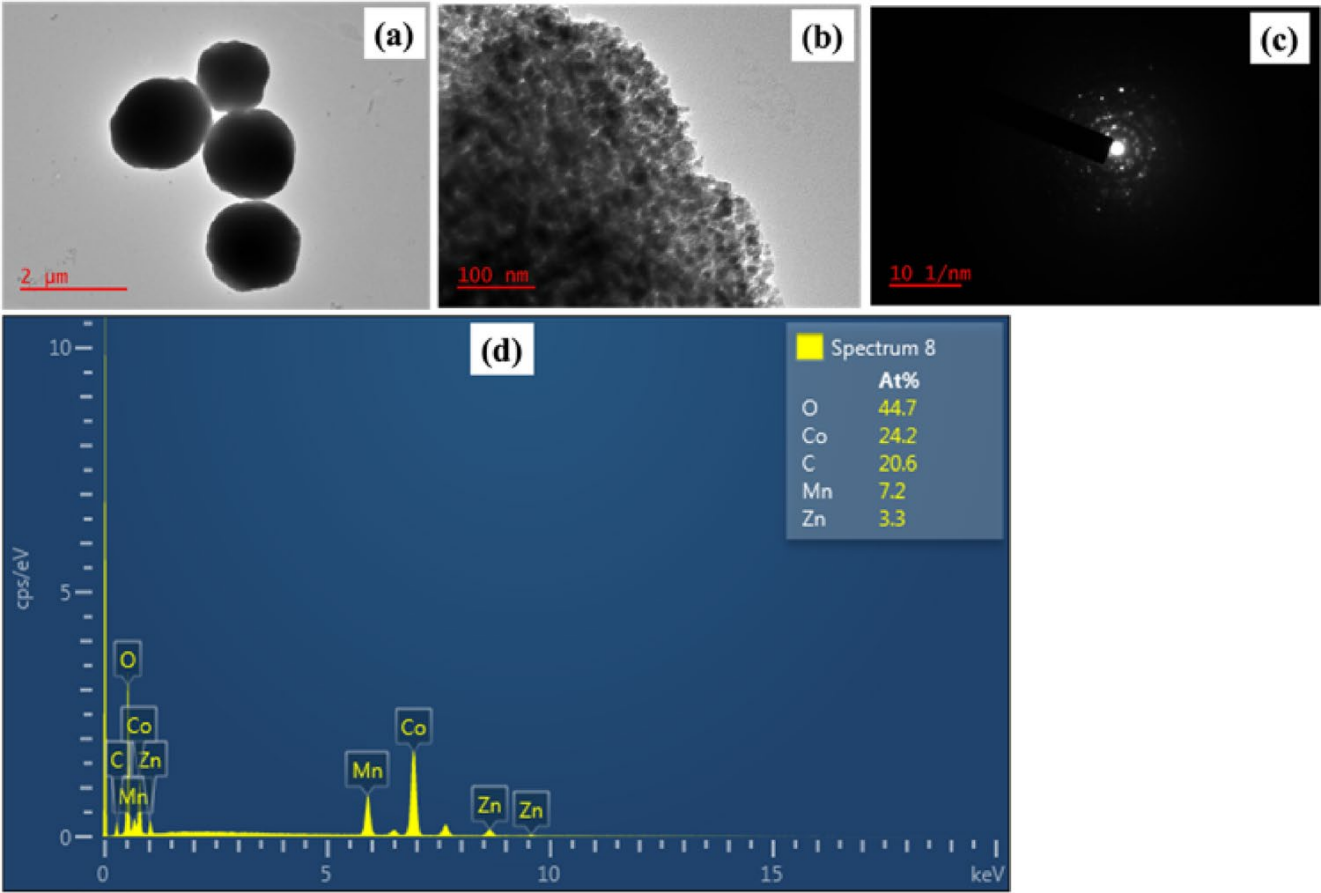
(**a**,**b**) TEM images at different magnifications, (**c**) SAED pattern, and (**d**) EDS spectrum of the ZMC3 oxide microspheres.

The morphology and microstructure of the Mn_x_Zn_1−x_Co_2_O_4_ microspheres were examined using scanning electron microscopy (SEM) and transmission electron microscopy (TEM). The SEM images in Fig. 2 depict ZMC oxide microspheres with diameters ranging from 200 to 600 nm. With the substitution of Mn in ZnCo_2_O_4_, more regular and abundant microspheres tended to form. Figure 3a presents a TEM image of the ZMC3 microspheres, which shows their smooth surface and micron size. In Fig. 3b, the high-resolution TEM (HRTEM) image zooms on the edge of the porous microsphere, revealing voids between the primary particles and a substantial quantity of nanoparticles. This porous structure promotes a relatively large contact area between the active material and the electrolyte, facilitating efficient ion transport^46^. Figure 3c shows the selected-area electron diffraction (SAED) pattern, which confirms the polycrystalline nature of the porous ZMC microspheres. This pattern also highlights the presence of abundant nanoparticles with a high phase purity. The EDS spectrum of ZMC3 in Fig. 3d indicates the presence of the Zn, Co, and Mn elements, with the Zn:Mn:Co ratio of 0.3, 0.65 and 2.2, respectively.

**Figure 4:**
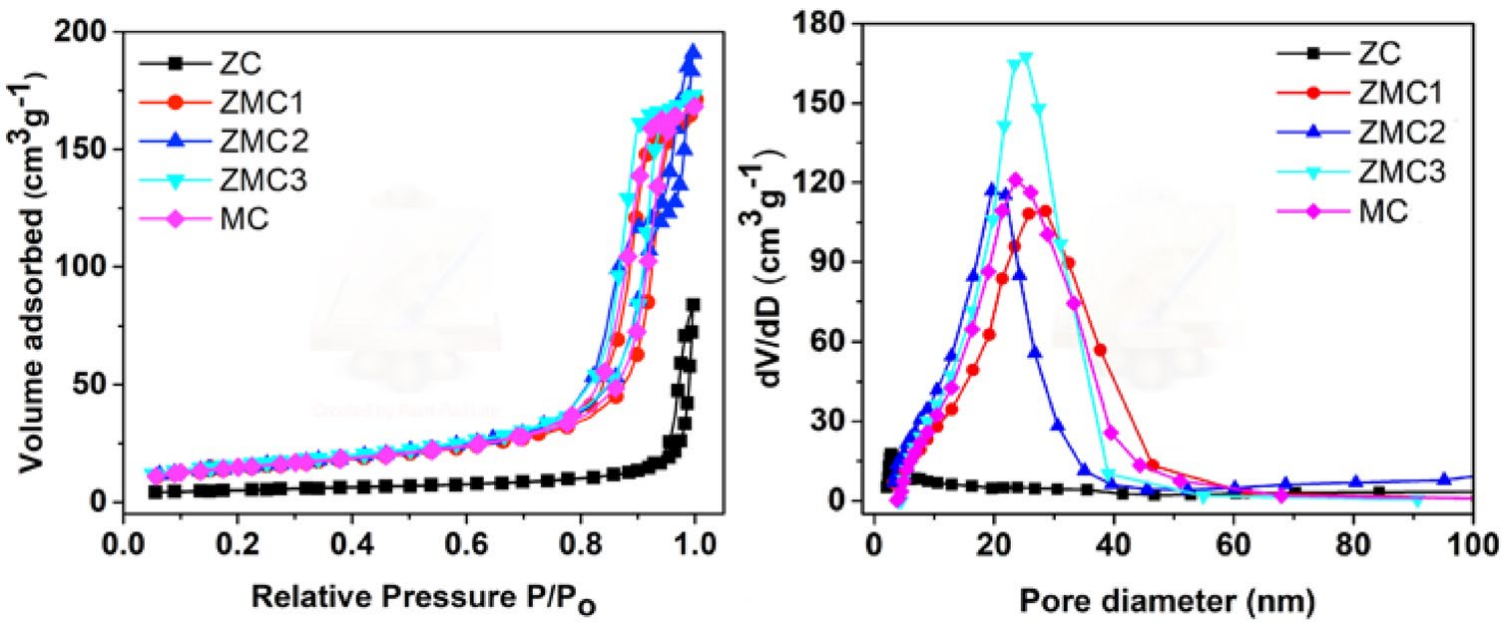
(**a**) Nitrogen adsorption/desorption isotherms and (**b**) pore size distribution of ZMC oxide microspheres.

The performance of a supercapacitor electrode is influenced by important parameters, such as the surface area and pore size distribution. To understand the textural properties of the as-prepared materials, nitrogen adsorption–desorption analysis was carried out and the values are summarized in Table 1. Figure 4a illustrates a type IV isotherm with a distinct hysteresis loop is observed at a relative pressure of 0.7–1 for all the Mn substituted ZMC samples. This indicates the existence of a mesoporous structure in the ZMC microspheres, which can improve the contact between the electrolyte and electrode and potentially reduce the diffusion paths for electrolyte ions. Incorporating Mn into ZnCo_2_O_4_ results in an increase in the surface area and pore volume. Specifically, the ZMC3 oxide microspheres displayed the highest specific surface area (56.27 m^2^ g^−1^) and pore volume of 0.29 cm^3^ g^−1^. This can be attributed to the open pores formed by the nanoparticles within the ZMC3 microspheres, as evidenced by the TEM results shown in Fig. 3b. Hence, it is anticipated that ZMC3 will exhibit superior electrochemical activity because of its large surface area and optimal pore size. The pore size distributions were determined by analyzing the desorption section of the isotherms, as shown in Fig. 4b. The results together with the summary of the values in Table 1 clearly demonstrated the presence of numerous mesopores ranging in size from 10 to 50 nm, indicating the simultaneous existence of structural and interconnected pores. Moreover, the pore size distribution curve revealed a prominent peak at approximately 23 nm, indicating a distinct mesoporous structure. BET analysis results confirmed that the substitution of Mn into ZnCo_2_O_4_ significantly enhanced the surface area, thereby contributes to an improved electrochemical performance of the electrode materials.

**Table 1:** BET surface area, pore volume, and pore size of the prepared materials.

Sample	BET surface area (m^2^ g^−1^)	Pore volume (cm^3^ g^−1^)	Pore size (nm)
ZC	17.80	0.00563	36.0
ZMC1	50.25	0.01280	19.4
ZMC2	51.47	0.01461	20.0
ZMC3	56.27	0.01353	17.5
MC	51.66	0.01171	18.4

### Electrochemical performance

**Figure 5:**
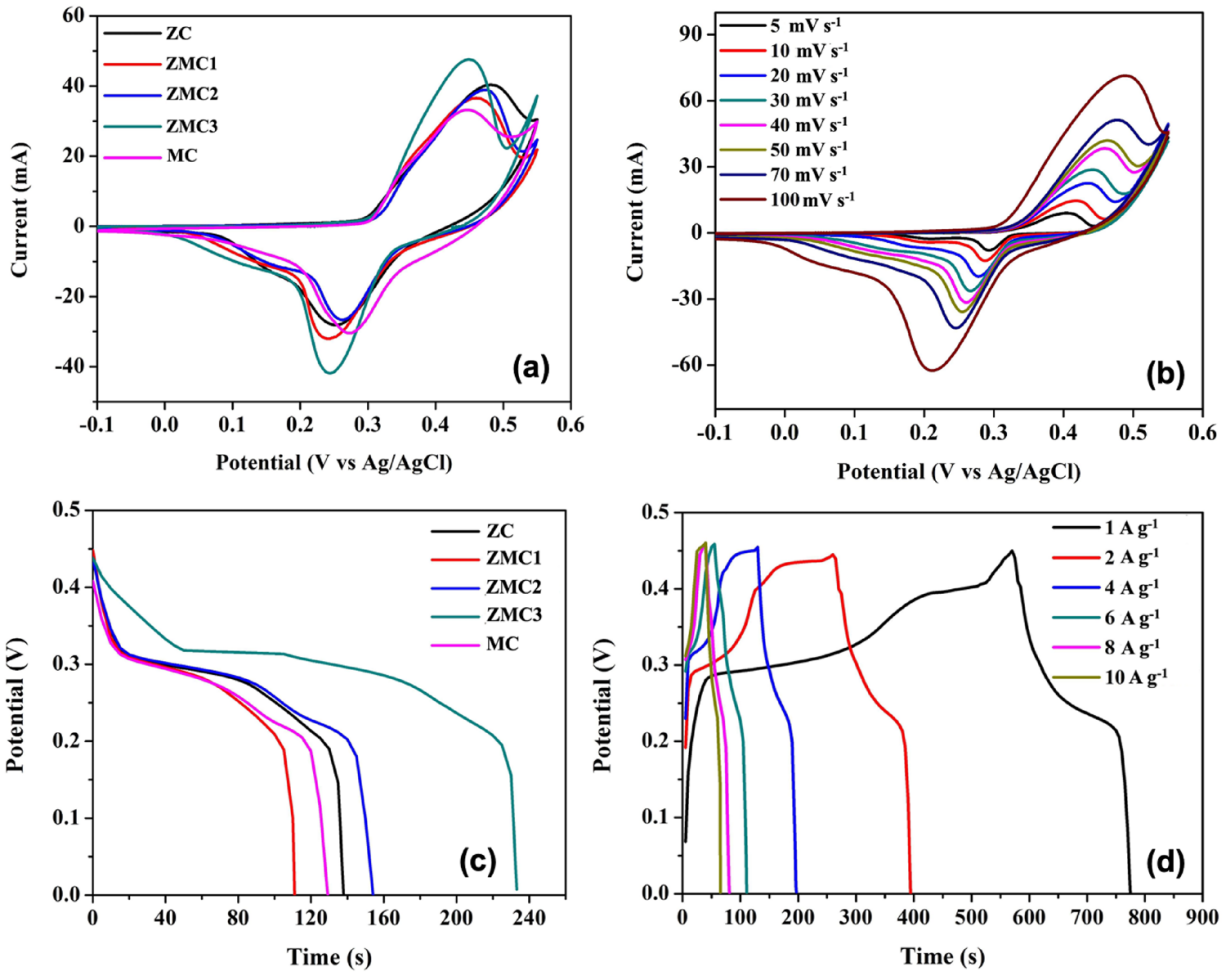
(**a**) Cyclic voltammetry (CV) curves of ZC, ZMC1, ZMC2, ZMC3, and MC at a scan rate of 50 mV s^−1^ in 2 M KOH electrolyte. (**b**) CV measurements of the ZMC3 sample at different scan rates from 5 to 100 mV s^−1^; (**c**) galvanostatic discharge curves of the ZC, ZMC1, ZMC2, ZMC3, and MC samples at a current density of 1 A g^−1^; (**d**) galvanostatic charge/discharge curves of the ZMC3 sample at different current densities.

The evaluation of the supercapacitive performance of the Mn_x_Zn_1−x_Co_2_O_4_ (ZMC) electrodes was performed by cyclic voltammetry (CV) and galvanostatic charge–discharge (GCD) measurements. All measurements were conducted in 2 M KOH electrolyte. Figure 5a displays the cyclic voltammetry (CV) curves of the ZMC oxide electrodes in a standard 3-electrode configuration utilizing the fabricated electrode as the working electrode, a platinum wire as the counter electrode, and Ag/AgCl as the reference electrode. CV was carried out at a scan rate of 50 mV s^−1^ within a potential range of − 0.1 − 0.55 V. The CV curves of all samples consist of pairs of well-defined redox peaks, indicating the presence of Faradaic redox reactions. This is quite different from the rectangular-shaped curves of the electric double-layer capacitance (EDLC), suggesting the establishment of the pseudocapacitive charge storage mechanism^47^. Among the samples, the ZMC3 sample displayed the highest redox peak current and largest integrated CV area, which can be attributed to the addition of manganese ions into the ZnCo_2_O_4_ spinel lattice^48,49^. Figure 5b shows the CV curves of ZMC3 at different scan rates. The shape of the CV curves remained consistent as the scan rate increased, indicating electrochemical reversibility and high-rate performance. However, there is a shift in the redox peaks towards lower potentials, which may be due to the polarization effect of the electrodes^50^. These redox peaks, originating from the Faradaic redox reactions, can be associated with M–O/M–O–OH (M = Mn, Zn, Co). The pair of redox peaks in the CV curves can be explained using the following equations^39,51,52^:

1 $${\text{ZnMnCoO + OH}}^{ - } {\text{ + H}}_{{2}} {\text{O }} \leftrightarrow {\text{ CoOOH + ZnOOH + MnOOH + e}}^{ - }$$

2 $${\text{CoOOH + OH}}^{ - } \leftrightarrow {\text{ CoO}}_{{2}} {\text{ + H}}_{{2}} {\text{O + e}}^{ - } { }$$

To further investigate the potential applications of the porous ZMC oxide microspheres as electrode materials for supercapacitors, we conducted galvanostatic charge discharge (GCD) measurementsat different current densities. Figure 5c illustrates the comparative galvanostatic discharge curves of the ZC, ZMC1, ZMC2, ZMC3, and MC electrodes at a current density of 1 A g^−1^ in the potential range of 0 to 0.45 V. The discharge curves show the pseudocapacitive characteristics of the as-prepared ZMC electrodes displaying a non-linearity and plateau regions. These features demonstrate the presence of Faradaic redox processes, and they agree well with redox peaks observed in the CV curves. A previous study on the Mn-substituted Mn_x_Zn_1−x_Co_2_O_4_ oxides carried out by our group utilized XPS analysis and revealed the presence of Mn^2+^, Mn^3+^, Co^2+^, and Co^3+^ in all compositions, which are important for redox reactions^44^. Consequently, the pseudocapacitive behavior observed in the electrode materials can be ascribed to the presence of Co^2+^/Co^3+^ and Mn^2+^/Mn^3+^ redox couples. This observation aligns well with the existing literature, which emphasizes the coexistence of Mn^2+^ and Mn^3+^ upon Mn doping, thereby enhancing the electrochemical performance of the host transition metal oxide electrode^42,48^. Moreover, looking more in to the GCD curve, it was observed that the ZMC3 exhibits a longer discharge time compared to other samples at the same current density and the same potential window. This indicated that the ZMC3 electrode exhibited a higher specific capacitance. For a more comprehensive understanding, the charge/discharge curves of ZMC3 at various current densities (1–10 A g^−1^) are presented in Fig. 5d. The specific capacitances C_s_ (F g^−1^) of the electrodes were calculated from the discharge curve using the following equation:

3 $${\text{C}}_{{\text{s}}} { = }\frac{{{\text{I }}\Delta {\text{t}}}}{{{\text{m }}\Delta {\text{V}}}}$$

where I (mA) is the applied current, Δt (s) is the total discharge time, m (mg) is the mass of electroactive material, and ΔV (V) is the potential drop during discharge. According to Eq. (3), the specific capacitance of the ZMC3 microspheres was determined to be 589.9 F g^−1^ at 1 A g^−1^. This value surpassed those of ZC (246.4 F g^−1^), ZMC1 (306 F g^−1^), ZMC2 (342.6 F g^−1^) and MC (286.7 F g^−1^) samples at the same current density. The increased specific capacitance observed in the ZMC3 sample can be attributed to its higher surface area and additional redox sites. These factors promote better interaction between the electrolyte and the active electrode material, facilitating more efficient electron transport. This electrochemical performance is comparable to that reported in the literature (Table 2). Mary et al.^41^ reported hydrothermally synthesized Mn-doped ZnCo_2_O_4_ nanoparticles with 1, 2, 5, 10, and 15% Mn doping by weight. The 10 wt% Mn-doped ZnCo_2_O_4_ sample achieved a maximum capacitance of 707.4 F g^−1^ at a current density of 0.5 A g^−1^. The presence of Zn, Mn, and Co in the Mn-substituted samples synergistically enhanced their electrochemical performances. In another study, Singh and colleagues explored the effect of Mn doping on the energy storage properties of Mn doped spinel FeCo_2_O_4_ (FeMn_x_Co_2−x_O_4_) nanofibers. The results demonstrated that Mn doping improved both the magnetic and energy storage properties of the FeCo_2_O_4_ spinel oxide, attaining a specific capacitance of 212 F g^−1^ at 3 mV s^−1^ and a Mn content of x = 0.2.

**Table 2:** Specific capacitances of ternary metal oxide-based electrode materials for comparison.

Electrode materials	Synthesis route	Specific capacitance	References
Zn-doped MnCo_2_O_4_	Coprecipitation	610 F g^−1^ at 1 A g^−1^	^53^
ZnCo_2_O_4_@MnCo_2_O_4_ nanosheets	Solvothermal	254 F g^−1^ at 1 A g^−1^	^54^
Mn-doped FeCo_2_O_4_ nanofibers	Electrospinning	191 F g^−1^ at 3 mV s^−1^	^55^
Ni-doped MnCo_2_O_4_ nanoparticles	Polymer-solution route	378 F g^−1^ at 1 A g^−1^	^56^
Co-doped MnFe_2_O_4_	sol–gel assisted hydrothermal	551.39 F g^−1^ at 1 mA/cm^2^	^57^
Mn, Zn co-doped NiCo_2_O_4_	Hydrothermal	513.2 F g^−1^ at 10 mV s^−1^	^58^
Mesoporous ZMC ternary spinel microspheres	Coprecipitation	589.9 F g^−1^ at 1 A g^−1^	This work

**Figure 6:**
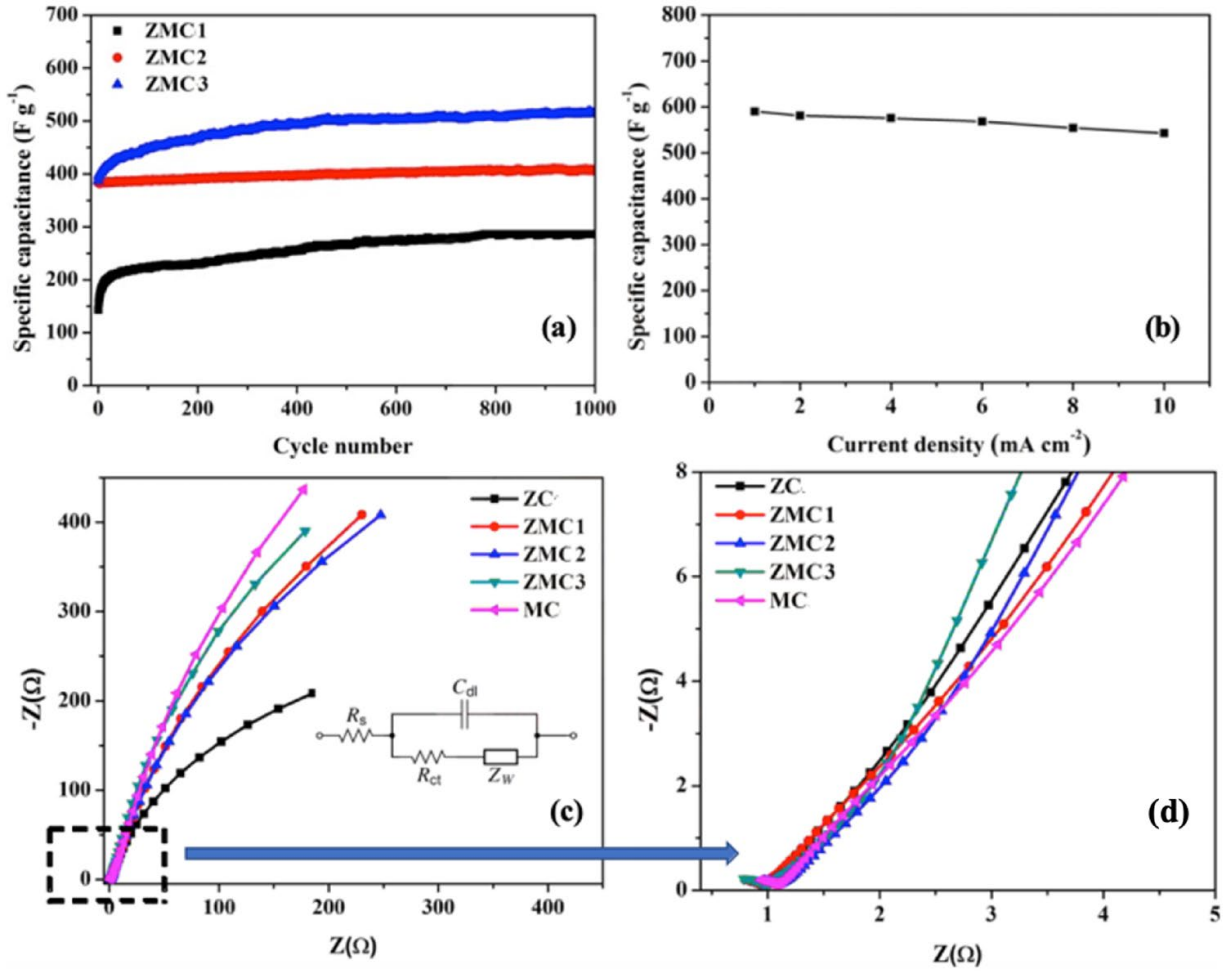
(**a**) Variation of specific capacitance with cycle number for ZMC1, ZMC2, and ZMC3 samples over 1000 cycles at a current density of 5 A g^−1^; (**b**) specific capacitance of ZMC3 electrode at different current densities; (**c**) Nyquist plots for ZC, ZMC1, ZMC2, ZMC3, and MC samples with an amplitude of 10 mV in 0.1 Hz to 100 kHz and the magnified portion of the higher frequency region. The inset in (**c**) shows the circuit used to fit the data. (**d**) Zoomed section of the Nyquist plots.

Supercapacitor electrode materials require cycling stability, which is a crucial factor for evaluating and comparing their performance with those of other reported materials. To evaluate the stability of the Mn-substituted ZMC1, ZMC2, and ZMC3 ternary oxide microspheres, they were subjected to 1000 charge–discharge cycles at a constant current density of 5 A g^−1^ in a 2 M KOH solution. The results are shown in Fig. 6a. The figure clearly demonstrates that the ZMC3 sample displays superior cyclic performance compared to the Mn-substituted ZMC1 and ZMC2 samples. After 1000 cycles, the electrode retained a specific capacitance of approximately 519 F g^−1^. In the first 450 cycles, the specific capacity increased from 383 F g^−1^ and subsequently stabilized, maintaining a stable capacity for up to 1000 cycles. Furthermore, when the current density was increased from 1 to 10 A g^−1^ (Fig. 6b), the specific capacitance of ZMC3 decreased to 533.1 F g^−1^, indicating a capacitance loss of only 7.9%. This result demonstrates the high rate capability at a high current density, which is a very important feature of the electrodes for practical applications.

EIS measurements were carried out to further investigate the electrical conductivity and electrochemical behavior of the electrodes at frequencies ranging from 100 kHz to 0.1 Hz with an amplitude of 10 mV. The Nyquist plots presented in Fig. 6c,d depict the characteristics of the ZC, ZMC1, ZMC2, ZMC3, and MC electrodes along with a magnified view of the high-frequency region. The inset in Fig. 6c shows an equivalent circuit diagram of the electrode materials. A typical EIS plot contains a semicircle in the high-frequency region and a straight line in the low-frequency region. The high-frequency region reflects the charge-transfer resistance (R_ct_) produced by the redox reactions that occur on the surface of the electrocatalyst. The low-frequency behavior reflects the diffusion of the electrolyte^59^. The Mn substituted Mn_x_Zn_1−x_Co_2_O_4_ electrodes exhibit equivalent series resistance (ESR) in the range of 0.96–1.1 Ω with ZMC3 providing relatively low overall resistance. The low ESR and charge transfer resistance for ZMC3 sample reveals its relatively high conductivity. The other major factor influencing the electrochemical performance of these materials is the alteration of the intrinsic activity of Mn-substituted ZnCo_2_O_4_.

As discussed in the preceding analysis, the enhanced electrochemical characteristics of the ZMC3 sample can be predominantly attributed to the synergistic influence of various multimetal compositions and the notably increased specific surface area achieved through Mn substitution^60^. The formation of mesoporous microspheres offers a high surface area accessible to electrolyte ions and enables faster kinetics and higher utilization of the active material. The substitution of Mn for Zn is believed to modify the distribution of cations across the tetrahedral and octahedral sites in the ternary spinel oxides, thereby fine-tuning their electrochemical properties and providing abundant electroactive sites^44^. In addition, the existence of various Mn oxidation states enhances the redox behavior of ZnCo_2_O_4_ by providing rich redox sites. While these results hold promise, a more comprehensive characterization and extensive electrochemical assessments utilizing two-electrode systems are essential to fully comprehend the intrinsic properties of these electrode materials and their potential for practical applications, which forms the next phase of this study.

## Conclusion

In summary, we prepared mesoporous Mn-substituted Mn_x_Zn_1−x_Co_2_O_4_ (x = 0, 0.3, 0.5, 0.7, 1) ternary oxide microspheres by employing a simple coprecipitation method followed by calcination. We systematically investigated the effects of Mn substitution on the structural and morphological evolution as well as the electrochemical performance of these materials. The XRD results confirmed the successful formation of spinel Mn-substituted Mn_x_Zn_1−x_Co_2_O_4_ ternary oxides, while the TEM and SEM images indicated the formation of microspheres with an enhanced specific surface area, providing more active sites for electron transport. The electrochemical activity of Mn_x_Zn_1−x_Co_2_O_4_ ternary oxides, when tested as an electrode material for supercapacitors, achieved optimum performance at a Mn concentration of x = 0.7. Notably, the ZMC3 electrode displayed the highest specific capacitance of 589.9 F g^−1^ at 1 A g^−1^ and retains 92.1% of its initial specific capacitance after 1000 cycles. Moreover, ZMC3 showed high capacity retention when tested at current density of 1–10 A g^−1^ indicating their remarkable rate capability. These findings highlight the significant improvement in the specific capacity and rate capability of Mn-substituted Mn_x_Zn_1−x_Co_2_O_4_ oxide microsphere electrodes achieved through Mn doping. Therefore, this study demonstrates the effectiveness of tuning the composition of multimetal transition metal oxides to significantly improve their electrochemical performance.

## Data Availability

The data that support the findings of this study are available from the corresponding author, upon reasonable request.
